# Young Schoolchildren’s Epistemic Development: A Longitudinal Qualitative Study

**DOI:** 10.3389/fpsyg.2020.01475

**Published:** 2020-06-26

**Authors:** Michael Weinstock, Vardit Israel, Hadas Fisher Cohen, Iris Tabak, Yifat Harari

**Affiliations:** Department of Education, Ben-Gurion University of the Negev, Beersheba, Israel

**Keywords:** epistemic understanding, epistemic development, interpretive theory of mind, knowledge justification, source of knowledge, discrepant claims

## Abstract

How children seek knowledge and evaluate claims may depend on their understanding of the source of knowledge. What shifts in their understandings about why scientists might disagree and how claims about the state of the world are justified? Until about the age of 41/2, knowledge is seen as self-evident. Children believe that knowledge of reality comes directly through our senses and what others tell us. They appeal to these external sources in order to know. The attainment of Theory of Mind (ToM) at this age is commonly seen as the significant shift in development in understanding disagreements in knowledge claims. Children attaining ToM understand that someone exposed to incorrect or incomplete information might have false beliefs. Disagreement, then, is still attributed to objective sources of knowledge. The current study examines the later developing Interpretive Theory of Mind (iToM) as the basis for children’s understanding of how people with access to the same information might disagree and what this means for how to provide justification for a knowledge claim. Fourteen 2nd graders with the most iToM responses to four tasks and 14 with the fewest iToM responses were selected from a larger sample of 91. In analyses of interviews about a story in which two experts make different claims about a scientific phenomenon, those in the high iToM group noted subjective perspective and processes as the source of disagreement and suggested the need for investigation as the means to knowing. In contrast, those in the low iToM group mostly could not explain the source of disagreement and held that knowledge is acquired from external sources. A comparison of the interviews regarding the science story 2 years later allows for a qualitative description of the development. Those in the low iToM group showed more general recognition of subjective and constructive processes in knowing whereas those in the high iToM group identified interpretive processes and the relativity of perspectives with implications for how observations were conducted and interpreted. Only those in the high iToM group referred to the importance of evidence as a basis for knowledge claims at either point in the study.

## Introduction

According to Kuhn (2001) and Kuhn et al. (2008), the separation and then coordination of theory and evidence are the essential bases for constructing claims in science and other knowledge domains. Epistemic understandings regarding the nature of theory and evidence as well as understandings of the standards of knowledge justification and the sources of knowledge underlie the competent performance of these tasks (Kuhn, 1991). Research has found that epistemic understandings are related to knowledge construction, justification, and evaluation among older school-age children (Mason et al., 2008; Iordanou, 2010; Barzilai and Zohar, 2012), adolescents (Mason and Scirica, 2006; Weinstock et al., 2006), and adults (Nussbaum et al., 2008; Kuhn et al., 2010; Weinstock, 2016).

Although younger school-age children’s epistemic understandings have been inferred from their behavior and responses to problems (e.g., Sodian et al., 1991; Pillow, 2002; Sandoval and Çam, 2011; Huang et al., 2019), with few exceptions (e.g., Ryu and Sandoval, 2012; Osterhaus et al., 2017) there has been little examination of their explicit epistemic understandings of theory, evidence, justification, and source of knowledge and of how they understand the epistemic characteristics of others’ thinking. Similarly, in research referring specifically to people’s understandings of the nature of science (NOS) there have been few studies on young school children and they tend to focus more on science reasoning, strategies, and use of theories and evidence rather than looking at their explicit understandings (Metz, 2004; Osterhaus et al., 2017). Koerber et al. (2015) did find that a significant percentage, although not a majority, of 9-year-olds did have understanding of at least one aspect of NOS. This shows that there is increasing understanding with age. But they also stressed that they could not identify a particular trajectory in the development of understanding of NOS. The current research is a step in trying to identify the trajectory of younger school-age children’s explicit epistemic understandings.

Although children’s verbal abilities might limit their meta-epistemic expression (Kloo et al., 2017; Huang et al., 2019), much research with adolescents and adults has shown that confronting discrepant knowledge makes epistemic perspective more salient (Perry, 1970; King and Kitchener, 1994; Kuhn and Weinstock, 2002; Barzilai and Weinstock, 2015). This seems also true of children as many theory-of-mind related tasks use competing claims or perspectives (Wellman et al., 2011; Osterhaus et al., 2016). The current research employs this discrepant-claims method to encourage children to express their epistemic assumptions. In short, we are interested in how schoolchildren understand and explain the source of disagreement between science experts and the means of resolving disagreement or uncertainty through justification. Although theory of mind (ToM) research is replete with examples of how children understand that different minds might know different things or how someone might lack knowledge (Wellman et al., 2011), we are interested in how theories of mind begin to be transformed into more generalized theories of knowledge, from understanding that someone with different information might have a false belief to understanding how it is that knowledge is not self-evident and that people might develop different claims based on the same information.

Much of the research on adolescents’ and adults’ epistemic thinking, such as the studies cited above, refer to a common description of epistemic development (Kuhn and Weinstock, 2002) which proposes a course of development through several epistemic perspectives. The developmental task that produces such development is to reconcile the objective and subjective aspects of knowledge. People progress from an “absolutist” understanding that knowledge is objective, certain, and in no need of justification, and has reality or authority as its source. With enough experience of uncertainty in knowledge and disagreement, even among experts, about what is known, people develop a “multiplist” understanding that there are multiple possible perspectives on truth. Knowledge is seen as subjective and basically uncertain because people do not agree with one another. Although there are means to explain one’s position, justification will not be fruitful because the source of claims are individual opinions, and thus, competing claims cannot be adjudicated. The succeeding “evaluativist” epistemic perspective is a shift from the radical, opinion-oriented relativism of the multiplist perspective while still understanding that knowledge may be relative to the knower and there may be different perspectives on truth. In this perspective, it is understood that judgments must and can be supported through processes of justification. It is understood that reality does not force knowledge on us, but that information must be interpreted in order to construct knowledge. Knowledge is not certain, but through the use of sound methods with clear standards of knowledge justification, one can be confident that an explanation is the best justified among possible alternatives. Experts are not seen as the ones who have simply gathered the most knowledge, or who assert their opinions the most persuasively. They are the ones who have used sound methods to generate evidence and provide reasonable interpretations. It is this evaluativist perspective which is seen as containing the understandings that allow for competent theory-evidence coordination.

Although there are no clear age ranges in which people obtain particular epistemic understandings, it is generally expected that absolutism is found more among schoolchildren, multiplism among middle adolescents, with some tendency toward a turn toward evaluativism in later adolescence and adulthood, particularly among those with higher education (Kuhn et al., 2000). However, in most age-group studies, all of the perspectives appear (Kuhn et al., 2000; Chandler et al., 2002; Barzilai and Weinstock, 2015). It has been argued that whereas the shift to understanding that there are multiple possible perspectives and relativism in knowing is a key developmental attainment (Chandler et al., 1990; Barzilai and Weinstock, 2020) there are several pathways that might follow this attainment including each of the general perspectives described above.

With that said, where does that leave research into the epistemic thinking of young school-age children? Even given the lack of clear age ranges in epistemic perspective development, there is some evidence (Kuhn et al., 2000) and little reason to believe that young school-age children will have made a turn toward the types of knowledge relativism represented by the multiplist and evaluativist perspectives. Indeed, ToM research has found that around the ages of 4–5, when children recognize that people might hold different beliefs, they still hold that the beliefs come from objectively gained information and that only one belief is true and others are false (Carpendale and Chandler, 1996; Wellman et al., 2011). Thus we expect that young school-age children will essentially be absolutist, believing in the objectivity and possible certainty of knowledge. Epistemic thinking researchers have referred to attainment of ToM, and its recognition that there may be more than one knowledge claim, as an important conceptual precursor to the epistemic development that is described above (Burr and Hofer, 2002; Kuhn and Weinstock, 2002; Iordanou, 2016). However, research has not shown the transformation of this important marker of epistemic development into more multi-faceted personal theories of the nature of knowledge and knowing.

The current study is part of a research project which is based on the proposal that it is not false-beliefs ToM, but a later development, interpretive theory of mind (iToM) which is the important first step in the developmental task of trying to reconcile and coordinate objective and subjective aspects of knowing, the task that drives epistemic thinking development. It is with iToM that the notion of objective and subjective aspects first becomes a clear issue in knowing, so we expect to see the first hints of a turn toward personal theories of knowledge accompanying iToM attainment.

Children’s attainment of theory of mind (ToM) is commonly assumed to be the watershed moment in understanding how people might assert different beliefs over the same matter of fact. In the false-beliefs task, 4–5-year-old children recognize that someone with incorrect information will form a different belief about an event than someone with correct information. For instance, a child with ToM who sees that a box of crayons actually contains candles will understand that someone who only sees the outside of the box will have a false belief about the contents of the box. (A child without ToM would claim the other person would know there were candles inside). Different claims are understood to arise when there are two sets of “facts,” one associated with a real event and the other with a different event mistakenly assumed to be real. In this sense, a child might recognize that knowledge is relative to the knower, but only because people have different information, which the child understands has been obtained through objective processes (such as seeing). However, how do children understand how people may make different claims about an event even when they have the same information? With iToM, attained at about ages 7–8 (Lalonde and Chandler, 2002; Osterhaus et al., 2016), children understand that people might disagree because they interpret the same information differently. With this, it appears that children with iToM recognize that a claim need not be self-evident, but that information can be used as evidence for more than one claim, and knowledge claims have subjective sources in the knower’s interpretation.

Specifically, we propose that ToM and the more advance iToM differ in how a child understands the knower and why people might assert different knowledge claims (Barzilai and Weinstock, 2020). ToM involves a type of perspective taking (about what someone knows or not given the information one has), and with the attainment of ToM children essentially hold that people know by using one cognitive function—perception—which they consider to be objective. In contrast, iToM does not involve taking a particular perspective but involves understanding that people do have subjective perspectives and make knowledge claims from these perspectives and may use a variety of cognitive activities (such as perception and interpretation). With other advanced, second-order ToM understandings children also understand that people gain knowledge through inference from information (Miller, 2009).

Although literature appears to point to attainment of simple ToM as a basis for children’s understanding of NOS (Koerber et al., 2015), in fact studies apparently showing a relationship between ToM and reasoning skills relevant to NOS actually have samples with children of an age (8, compared with 4–5 when ToM is attained) in which it might be assumed that they have iToM or other more advanced theories of mind (Pillow, 1991; Carpendale and Chandler, 1996). Indeed, Koerber et al. (2015) do conclude that it seems that it is the children with more advanced theories of mind (at age 9) that demonstrate better understanding of NOS. The same researchers (Osterhaus et al., 2017) found that a measure of advanced ToM, which did not include iToM, did predict epistemic understanding of NOS.

In the current study, we explore how responses to the simple test of iToM—in which children with iToM claim that two people might have different interpretations of ambiguous pictures—might be represented in a more complex representation of interpretations of ambiguous evidence for scientific claims. Rather than just displaying an understanding that two people might produce different interpretations of the same picture, the children are asked to explain how two science experts might arrive at different conclusions about a biological phenomenon. As part of this research project, a preliminary quantitative analysis has found that indeed iToM, but not ToM, predicts epistemic understandings of the source, structure, and justification of knowledge that reflect the first grappling with subjective aspects of knowing within the absolutist perspective (Weinstock, 2018). The current analysis, however, is not concerned with tracking advancements in the epistemic perspectives of the developmental model (Kuhn and Weinstock, 2002). Rather, here we use a qualitative approach to describe the epistemic growth of children at two points in time looking at their expressions of issues such as perspectives and interpretation in the knowing process, the reasons for disagreement among experts, understandings of how knowledge is constructed and justified, and the nature of evidence. In addition to this developmental description, we assume that those demonstrating iToM at an earlier age, having understanding of subjective processes of knowing as a basis for knowledge claims, will show more advanced understandings of the epistemic issues across the 2 years of the study.

## Materials and Methods

### Participants and Selection Criteria

This study sample was selected from a larger sample of 91 2nd graders (*M*_*age*_ = 7.05, range = 7.42–8.75) from two urban elementary schools who participated in a longitudinal research study that also included kindergarteners and 1st graders. All of the children were assessed three times at 1-year intervals. The current analysis includes a selection of these participants from the first and last years of the study, that is when they were in 2nd grade and then again from when they were in 4th grade. On the basis of responses to the assessments (to be described in the results section), the data of 28 2nd graders were included in the first wave and of 11 of these children when they were in 4th grade.

The population from which the sample was drawn was chosen to represent average Israeli children: The city is rated at the higher end of middle class (Central Bureau of Statistics, 2017), and each school has essentially average scores on a system-wide standardized test of academic skills (Ministry of Education, 2013). Among countries, Israel is ranked as having very high development (Human Development Report Office, 2015).

The research was approved by the Ministry of Education and the university human subjects research committee. The parents of each of the participants consented to the participation of their child. In addition, on the day a child was scheduled for the research, the child first gave consent to leave the classroom and then signed a consent form to participate in the research that was read aloud by a research assistant.

### Materials

In each wave of the study, the children were assessed for iToM. They also responded each year to an epistemic perspective assessment in which two experts disagree about a biological phenomenon. This was immediately followed up by a semi-structured interview prompting reflection about the nature of knowledge and knowing, the reasons people might disagree about knowledge, and the components of knowledge justification. The semi-structured interview was the basis for the current, primarily qualitative analysis. Nevertheless, we describe the iToM task because we used this to select the sample for the current analysis as we explain in the results section. We also describe the epistemic perspective assessment because participants sometimes referred to it in their responses to the interview, so it is important to provide the context. All of the tasks and interviews were presented in one-on-one sessions with the researchers or research assistants in separate rooms. The sessions lasted between 30–45 min.

#### Interpretative Theory of Mind (iToM) Task (Lalonde and Chandler, 2002)

The participants were given the “droodles” task (Lalonde and Chandler, 2002) to assess for ToM and IToM. The task involves presenting a child with a picture and having the child describe the content of the picture. The child is then shown the same picture but framed in such a way that only an ambiguous portion of the picture is visible. The child is then asked to state what two different people (represented by adult-looking dolls) would say the picture they see is of. A child who states that the first doll would say that the picture is of what the child originally saw when looking at the whole picture is not considered to have achieved ToM (as this is essentially a false-beliefs task). A child who states that the first doll would say that the picture is something different from the original, whole picture would be considered to have passed the false-belief ToM task, as that child understands that the doll has different information than she or he does. But only if the child then states that the second doll sees something different from the first doll would the child be considered to have iToM in that the child recognizes that two people might have different interpretations of the same information. A child without iToM would insist that the two dolls must see, and know, the same thing as each other even if they do not know what is in the full picture.

There were four pictures. A point was given for each picture that a participant gave an iToM response to. Thus, each participant had an iToM score of 0–4.

#### Epistemic Perspective Assessment

An epistemic perspective assessment was developed for use with children at this age based on the format of discrepant accounts assessments used with older ages (Kuhn and Weinstock, 2002). The assessment is in the form of a picture book that tells the story of a new creature on a foreign planet. The prince/princess of the planet wants to understand what the creature uses its unusual hands for in order to explain the phenomenon to the king who has not seen the creature. Two expert advisors give conflicting explanations. One says the creatures has large hands with sharp nails to dig for food, and the other says these types of hands are used to hang on to tree branches when sleeping at night. During the course of the story, the participants were asked why the two experts did not agree, what they would do to decide on an explanation of the phenomenon, and how someone could decide about the correctness of a third person’s claim about the phenomenon.

Analysis of responses to questions asked during the reading allowed for coding in levels that were conceptually consistent with three types of absolutism (realist, pre-dualist, and dualist), multiplism, and evaluativism (see Weinstock and Cronin, 2003) in three epistemic dimensions of source, justification, and structure (simplicity/certainty) of knowledge (Hofer and Pintrich, 1997).

#### Reflective Interview

The epistemic perspective was immediately followed by the more general questions:

(1) Is it possible for two people to see exactly the same thing and think different things from each other?
(2) Can you explain to me what knowledge is? What does it mean to know something?
(3) How do we know things about life?
(4) What is the difference between knowing and guessing?
(5) If you do not know something, what can you do to know it?

Although just a limited number of interviews from the total sample were used for the current analysis, all the participants responded to this interview. In order to understand the response and develop themes, we read a larger number of interview responses than were ultimately used in the analysis. The coding scheme for the reflective interview was developed and employed with a series of iterative, collaborative processes. The goal of the researchers was to develop shared, describable representations of themes across interviews and categories of thinking within those themes.

One of the project researchers selected 10 of the interviews for use in developing the coding system which were ultimately not used in the final coding. The participants were selected to represent a range of iToM scores. The researcher who selected the interviews was not involved in their coding, and the two researchers developing the coding system were blind to the iToM scores.

As a first step in developing the coding system, the two researchers randomly selected five of the 10 interviews. They read each interview in full and, together, worked to make sense of the conceptions about knowledge and the processes of gaining knowledge that were expressed by the interviewee. After discussion, the researchers outlined the epistemic conceptions presented by each interviewee. Next, the researchers sought connections between conceptions of different interviewees on the same topic. In this way, the researchers defined themes that seemed to be repeated across interviews.

In the second step, the researchers worked separately to code the remaining five interviews based on the themes identified in the first five interviews. In this phase, the researchers’ coding process was deductive, in analyzing the interviews based on the themes developed in the first step, and inductive in identifying expressions that extended the existing themes or suggested new themes. After coding three of these five interviews, the researchers compared the codes and resolved any differences or open questions. After comparing their separate analyses of the remaining two interviews, the researchers found that their codes were mostly similar and they referred to the same statements in support of the themes.

With this level of confidence, the coding system was formalized with a table of themes which were to be used to make a first pass at analyzing the 28 interviews from the 1st year of the study used in the current analysis. The 28 interviews were selected by a researcher not involved in the coding based on iToM scores. Fourteen of the participants had the very highest number of iToM responses, and the other 14 had the very lowest number of iToM responses. It should be noted that those developing the themes and coding these interviews according to them did not know which participants were in the high and low groups.

In this phase, the researchers elaborated detailed definitions for the themes while establishing them with quotations from the children. The inductive search for additional themes was continued throughout the coding, but the researchers verified that this system did indeed cover and exhaust the codes afforded by the data from the wide range of participants. This indicated that the coders had achieved inductive thematic saturation (Saunders et al., 2018) in developing the coding scheme.

At this point, the researchers began the deductive coding process of the 28 reflective interviews. This process was also undertaken in stages. First 10 of the interviews were randomly selected for coding by each of the researchers. They compared the coding of each interview and documented instances in which there were disagreements about how certain types of expressions would be coded. These disagreements were resolved and the documentation allowed for more elaborated and differentiated definitions of each theme. We followed the approach of achieving reliability through discussion and consensus in order to maximize the refinement of understanding of the coded ideas (Barbour, 2001; Campbell et al., 2013; Gläser-Zikuda et al., 2020). This process was repeated until all of the interviews were coded.

Whereas eight themes were developed, the current research focuses on two of them: (a) the dawning understanding of subjectivity as an explanation for how disagreements about knowledge arise, and (b) how one gains knowledge. The four different response types of how disagreements arise are described briefly here with coding examples given in Table 1. (1) Some children did not recognize or ignored discrepancies between the accounts of the scientists in the epistemic perspective assessment, and their discussion of the possibility of discrepant claims reflected this. They insisted there was and could only be a single, objective claim. Whereas most participants recognized discrepancies and acknowledged that people might claim different things even if they had the same information, (2) some acknowledged this without providing any explanation, (3) some explained this as simply because people are different so their minds have different content, and (4) some offered reasons concerning different perspectives on information or different ways of thinking. It should be noted that in the original coding scheme there was an additional response, categorized between types 2 and 3. It was characterized as acknowledging differences with the explanation that people have different perceptual experiences. However, this response was seen in just one of the initial protocols used to develop the coding scheme, and in none of the protocols of the 28 participants analyzed for this study.

**TABLE 1:** Category coding for the theme of explanations of how disagreements about knowledge arise.

Category	Characteristics	Examples
Single, objective claim	Competing claims not recognized as possible or not acknowledged.	Gave simple “no” in response to probe: “Is it possible for two people to see exactly the same thing and think different things from each other?” No spontaneous reference to competing claims in the remainder of the interview.
Different claims, no reason given	That people might make different claims is acknowledged but without any clear explanation or elaboration of why such disagreements might arise.	“It came out that way, because it might have come out that [it is different].”
People are different, having different minds with different content	Discrepancies explained by noting that people are different. They think differently because they are different. No elaboration on why people being different with different minds might lead to holding different knowledge.	“Let’s say there’s a fire somewhere, they’ll think, one would think it’s because there was too much heat, and one might think somebody lit a fire there and it spread. Everyone sees it differently.” “Everyone thinks what he knows and thinks.”
People have different perspectives, ways of thinking	That people have different lived lives, perspectives, and ways of knowing is the source of how they might arrive at different claims.	“It may be that Rona lives in a very large house and that is why this house looks small to her and Karen lives in a small house and now it looks big to her, so they can argue, because everyone thinks something different”

The response types are presented in order from objective to subjective, and within the subjective from less to more elaborated in explaining the source of the disagreements in terms of subjective processes of the knower. Ultimately, for the sake of comparison in the analysis, each participant was assigned to a response category based on their highest level of explanation.

There were three response types regarding how one gains knowledge (see Table 2 for coding examples). Essentially, there were responses that referred to objective sources of knowledge—(1) perception or (2) external authority—and responses that referred to (3) aspects of individual knowledge construction, particularly investigation, although not much to interpretation, as the source of knowledge. For the sake of comparison in the analysis, participants were assigned to response types according to dominant answer. None of those assigned to response types 1 or 2 (i.e., both representing knowledge as gained passively through external sources) gave responses consistent with type 3 (knowledge gained through active construction).

**TABLE 2:** Category coding for theme of understandings of how knowledge is gained.

Category	Characteristics	Examples
Senses, direct perception	Knowledge gained through the senses	“If you know something, you must have listened to something or seen.”
External authority	Knowledge is learned, passively acquired from authoritative external source, presenting information, such as teachers, parents, friends, books, or the internet.	“…learn, learn. from school or high school or anywhere.”
Investigation, exploration	Knowing involves intentional activity on the part of the knower. Goal-directed investigation, exploration, and questioning are needed to confirm and develop ideas. Might accept others as ultimate objective authorities, but knowing requires effort on the part of the knower.	“Knowledge is when you research something and you know it really already, without mistakes.” “There are many methods. Go and ask, do research….Come to class and say I am in kindergarten and I want to know what it is like to be in first grade and then you are told. You ask a child to bring all the school-related things: a symbol, a set of hours, everything and then he gives an explanation.”

Certain themes, such as these two, emerged clearly in the words of all the children in response to questions in the interview. Because they were easy to identify in all the children these themes suggested a conceptual sequence and it was possible to place each child’s responses in that sequence. Moreover, understanding the possibility of multiple, disagreeing perspectives is seen as a foundational aspect of epistemic development (Kuhn et al., 2000; Barzilai and Weinstock, 2020), as is understanding how one gains knowledge (Miller et al., 2003; Pillow, 2008; Fitneva et al., 2013).

The other themes (checking the truth of a knowledge claim, general understanding of the sources of knowledge, conceptions of expertise, conceptions of absolute truth, certainty, and the difference between guessing and knowing) are also informative regarding the epistemic thinking of children; that they were expressed spontaneously in some, but not all participants, shows that at least some children at this age attended to such issues. However, for the sake of the current longitudinal analysis they will remain in the background. These themes tended to represent specific issues subsumed in the broader themes of why there are disagreements and how people come to know. Moreover, several of these themes, such as checking the truth of knowledge claims, were not expressed by all of the interviewees. On one hand, the lack of expression of these themes may be meaningful in that this could indicate that the children had not yet developed concepts regarding such issues (i.e., the children may not have considered that knowledge claims need to be verified), or no longer held the concepts (i.e., that knowledge is absolute). On the other hand, it is also possible that the interview questions simply did not sufficiently probe these issues. In either case, these themes could not be found consistently expressed by all participants in the interviews conducted in the first and last years.

## Results and Discussion

As mentioned above, the responses to 28 semi-structured interviews from grade 2 were chosen according to level of iToM exhibited by the children. Because of the demands of coding the interviews, and because the major goal of the analysis is to describe the range of epistemic development rather than focus on hypothesis testing, the interviews were selected according to criteria rather than randomly to try to capture a range of epistemic thinking. The selection criteria at grade 2 and grade 4 were used in order to be able to develop a comprehensive picture of epistemic development over 2 years leading from iToM attainment. The reflective interviews from grade 2 chosen for analysis included those from participants with the highest (*n* = 14) and lowest (*n* = 14) number of iToM responses. Eleven of those reflective interviews were selected for comparison with those participants’ reflective interviews from 4th grade. The 11 were selected according to responses they had made in 2nd grade so that the development from a range of responses could be described across the 2 years. The interviews of these 11 participants were used for the longitudinal comparative analysis between 2nd and 4th grade presented here. The themes developed at the first wave were further elaborated and refined for this analysis by one of the researchers who had been involved in developing and employing the coding scheme in the first wave. The refinements included expressions that were found in the discussions in developing the themes with the grade 2 interviews but were not frequent enough to warrant distinction from the categories found. At grade 4, with greater salience, it became possible to make more specific distinctions.

The interviews from 2nd grade were analyzed to identify overall epistemic themes. As described earlier, two themes were identified consistently across participants: understanding why disagreements arise about knowledge claims and understandings of how knowledge claims are constructed and justified. The first theme has particular importance because it reflects the consideration of subjective perspective in knowing which is the issue which we assume is the essential proposed connection between iToM and further epistemic development. We used this classification of responses in describing the participants in the comparison between 2nd and 4th grade as we were interested in tracking shifts toward relativist thinking. Before analyzing the comparison between the grades we present the distribution of participants across response types in these themes from 2nd grade in order to illustrate their relationship to iToM attainment.

### 2nd Grade Interviews

There were four types of responses reflecting understanding of the source of discrepant claims that the participants gave, primarily to the interview question, “Is it possible for two people to see exactly the same thing and think different things from each other?” However, other places in the interview where the participant mentioned the disagreement between the scientists in the story were also taken into consideration. The response types are listed in Table 3 in order of our assumptions about epistemic development (Kuhn and Weinstock, 2002). (Refer to Table 1 for the definitions and examples of each response type). Table 3 shows the distribution of these response types by high and low iToM response. Half of the analyzed sample was chosen because they gave no or the fewest number of iToM responses in the iToM task while the other half had given the highest number of iToM responses.

**TABLE 3:** Explanations of how disagreements arise about knowledge by iToM response level.

Discrepant claims explanation	iToM response level	
	Low iToM (*n* = 14)	High iToM (*n* = 14)
Single, objective claim	1	0
Different claims, no reason given	5	1
People are different, different minds with different content	7	10
People have different perspectives, ways of thinking	1	3

As can be seen in Table 3, those with low iToM tended much more than those with high iToM to state that there was one objective claim that was correct or did not give reasons why the scientists had given different claims. In contrast, those with high iToM almost entirely focused on the subjectivity of the scientists, saying they were different people with different ideas or that they had reasons or backgrounds to take different perspectives in making their claims. Just to confirm this pattern, a 2 × 2 Fisher’s exact text, which can be used with small samples, was performed with not recognizing or not finding reasons for discrepant claims in one group, and those acknowledging subjective states or processes being in the other group. The test was significant, *p* = 0.038.

Table 4 shows the distribution of response types regarding understandings of how people gain knowledge. (Refer to Table 2 for definitions and coding examples). These responses were to the interview question, “If you do not know something, what can you do to know it?” However, other places in the interview where the participant mentioned the source of knowledge were also taken into consideration. As can be seen in the table, almost all with low iToM gave one of the objectivist responses. Those with high iToM were split among the objective and constructive response types, but almost all of those expressing constructivist ideas were those with high iToM. Again, a 2 × 2 Fisher’s exact test showed an association between level of iToM and response type, *p* = 0.038.

**TABLE 4:** Understandings of how knowledge is gained by iToM response level.

	iToM response level	
Knowledge sources	Low iToM (*n* = 14)	High iToM (*n* = 14)
Senses, direct perception	2	1
External authority	11	7
Investigation	1	6

### 2nd-4th Grade Comparisons

For the comparative analysis between 2nd and 4th grade, the intent was to choose the interviews from both grades of four children from each the “different claims, no reason,” “people are different/have different minds,” and “people have different perspectives/ways of thinking” categories to examine the trajectories of development from those starting places. As one of the children from the “different perspectives” category had dropped out of the study by the last year, and there were only three left in this group, the total sample was 11.

#### Understanding of Disagreements Between Knowledge Claims

For the issue of understanding of how disagreements about knowledge can arise, the participants’ responses at grades 2 and 4 are presented here in pairs in order to illustrate the development. The cases we are presenting were chosen to be representative of the refined, more specific themes developed in this analysis. For each participant, the iToM categorization and the categorization for understanding of sources of disagreement in 2nd grade are noted. The understanding disagreeing claims categorization in 4th grade is also noted. The first finding, which will be illustrated, is that the different perspectives and ways of thinking category was differentiated. Some responses noted that there were different perspectives and ways of thinking while another response type, which is apparently more advanced, specified internal thinking processes as the source of individual’s knowing that could result in disagreements. Although we do not present all of the 11 cases, it should be noted that in all cases, except for those who had already expressed understanding of internal processes in 2nd grade, there were epistemic shifts toward more consideration of the subjective aspects of knowing from grade 2 to grade 4.

The first comparison includes two cases of participants who had low iToM in 2nd grade, and who were responding to the question, “Is it possible for two people to see exactly the same thing and think different things from each other? Why/why not?” In each case, in 2nd grade the participants imply that subjective differences can be overcome so disagreement is not necessary (coded as different claims noted, but giving no reasons to explain this). By 4th grade each acknowledges that subjectivity is at the root of knowing, but with a focus only on opinion or motivation with no mention of interpreting information differently (coded as different perspectives or ways of thinking). For instance, as a 2nd grader P116 states: “Because it’s not the same people and they do not know each other” with the implication that if they did know each other they would not have disagreed or they could work out their disagreement. In either case, there is no explanation of why not knowing each other would lead to disagreement between people. In 4th grade, P116 states: “Everyone has her own opinion” giving a reason for the disagreement and focusing on subjectivity. The stress on opinion is the hallmark of multiplism, the most subjective epistemic perspective (Kuhn and Weinstock, 2002).

Similarly, 2nd grader P74 says: “Because he doesn’t not need to know what I know, If I tell him then he would know.” Although approaching the different people have different minds with different content category, in this case the participant diminishes the effect of this and does not explicitly explain how this should lead to different claims. The tone of P74’s statement is that one person knows and the other does not, but could know. It is not that the minds have different content, but one mind has more information than the other. In response to a question from the epistemic perspective assessment about how it could be that the expert royal advisors had two different answers, 2nd grader P74 said, “Because they did not hear each other they think differently.” That is, as long as people manage to have the same information, they will agree. At 4th grade P74 does give an explanation for differences rooted in subjectivity, “Because we are not the same person, if we were thinking the same thing it would be boring.” But, in response to the epistemic perspective assessment questions, in 4th grade P74 still thinks that subjectivity is just a distraction and that ultimately different knowledge claims can be adjudicated by sharing information: “In the end they will reach a decision and they both will be right. They’ll say: Let’s go and see.”

Similar to the opinion and motivation oriented reasons given in 4th grade by P116 and P74, a participant (P249) who had high iToM in 2nd grade said this in 2nd grade: “Everyone has their own opinion, not everyone has the same opinion.” By 4th grade, this participant focuses in a range of internal processes and bases for multiple perspectives, of which opinion is just one:

“There can be a situation like this, as we learned about the two characters who managed to discover two different things from the same part of a picture [from the iToM task]. Everyone can hear a different side and another place, another story and another opinion.”

Rather than just throwing out the notion that people have different perspectives, P249 specifies what perspective might mean and hints at the implications of people holding different perspectives. P144, who also was high iToM and started out in 2nd grade straightforwardly expressing the different ways of thinking response, although with no mention of opinion—“Everyone thinks differently”—also further specified the internal processes of thinking that could produce discrepant claims. In 4th grade he said, “Everyone has his imagination, his world, and thinks differently.”

From the examples given so far, we can see that some of the participants seemed to be working out the role of subjectivity in knowing. This trend started with the acknowledgment of subjectivity without any clear explanation of its necessity or why it played a role. This advanced to the unavoidability of subjectivity, especially with reference to opinion. Then a few specified subjective ways of thinking beyond opinion, but with a focus on idiosyncratic characteristics as the source of perspectives.

In another trend, the development moves toward consideration of processes of interpreting and emphasizing information and is more empirically oriented. That is, there is more of a focus on knowledge issues than character issues, although character issues still come into play. There are also explicit and implicit references to how fields of expertise or interest influence ways of thinking. This can be seen in the responses of two participants who were high iToM and were categorized as focusing on internal processes as the reason for disagreement at both grades 2 and 4. In 2nd grade, P151 expresses internal processes somewhat in line with the emphasis on idiosyncratic characteristics and preferences: “There is a Lego that one likes and another doesn’t, so everyone says something different about it.” In 4th grade, there is a turn from preferences to attributing disagreements to fields of expertise and the subsequent effect on thinking: “Because they seem to be experts on different things so they will think differently.” 2nd grader P180 starts with a seeming reference to expertise and its relation to ways of thinking, but defining it as a matter of preference. She said:

“There are people who like tree tops and some who love plants so maybe they explain different things because they think that way.”

She still focuses on preference in 4th grade, but unlike the previous trend she mentions empirical implications.

“Everyone has a different character and a different look, and each one sees from one’s angle. One will see fingernails on the little finger and the other on the thumb.”

With this, there is developing appreciation of expertise and perspective that it brings. In response to the question from the epistemic perspective assessment about how it could be that the expert royal advisors had two different answers, 2nd grader P248, who had low iToM and did not give reasons to explain why people might disagree, simply said: “Because neither of them knows.” By 4th grade, he said, “Both are correct. One is an expert on trees and one is an expert on land.” This is consistent with his response to the reflective interview in which he said that disagreement exists because people have different perspectives and ways of thinking. People might disagree not just because of idiosyncratic perspective and opinion, but because of emphases in what they know and how they explain given their field of expertise. P249, who had high iToM had a similar response to the same question, but in 2nd grade. “I think because each one is an expert on something else and each thinks of something she is an expert in.” In 4th grade, P249 expresses how perspective complicates the process of knowing. “That implies one does not always come to an exact answer. This is problematic.” This view, that some uncertainty and tentativeness is part of knowing, is decidedly not absolutist.

#### Understandings of How Knowledge Is Gained

The children in this study had a harder time expressing what they think of how knowledge claims are constructed and justified, even in 4th grade. This is presaged by the coding of their understandings of how knowledge is gained from their 2nd grade interviews as outlined in Table 4. Just one person with low iToM referred to investigation as a way to gain knowledge, as did minority of even those with high iToM. In either 2nd or 4th grade, those with high iToM did tend to indicate the need to develop a base of evidence in response to the reflective interview questions (i.e., “Can you explain to me what knowledge is? What does it mean to know something?”, “How do we know things about life?”, “What is the difference between knowing and guessing?”, and “If you do not know something, what can you do to know it?”). However, as will be seen, their ideas of what counts as good evidence is not always clear, although in contrast to those with low iToM they did indicate the need for evidence. The examples given only include those with high iToM, because those with low iToM simply did not refer to evidence. (One notable characteristic was that several told fantastical stories about the creature that were not even based on the information about the creature in the story book).

To give an example of what developed, in response to the question about the difference between knowing and guessing, P249 gave this answer as a 2nd grader in the first year of the study:

“When I guess something, I’m not sure, but a guess a hypothesis. ‘Knowledge’ – if you’re still learning, you still do not know everything. There are all kinds of answers, and there’s a chance you’ll answer that you did not learn about it and you will not know the correct answer and you will be wrong.”

Whereas this participant suggests that multiple answers must be considered, it seems that the participant regards knowledge as being mostly certain whereas a guess is not correct and is wrong. As was expected with iToM, there is a suggestion of subjective processes in knowing, in particular in coming up with the wrong answer. However, there is no mention of evidence. But in the third year of the study when the participant is a 4th grader, the participant does see that knowing requires some type of evidence.

“To know something is to be certain of something. that you have evidence, and you saw it in your eyes. A guess is a hypothesis of something you cannot be sure of and you think about.”

Although this participant does say one needs evidence, the idea of evidence remains at the level of personal observation. Other participants with high iToM offered more complex views of evidence. For instance, in response to the same question, P151 said as a 4th grader in year 3 of the study:

“He is going to see, and was confirmed by several witnesses. Everyone comes because he can lie…. Anyone can imagine differently (from the other) and then check who was right.”

Although not directly talking about justification, this participant does say that claims need to be supported by evidence, and that reports of evidence and claims need to be judged and justified.

Also in response to the question regarding the difference between knowing and guessing, P144 said:

“Knowing that you know more about this and that is pure. You saw, you read, you heard. To guess, you just think about it with your head.”

In this, the participant mentions that to know one needs multiple sources of evidence, and notably, not just evidence based in direct observation. We also see here indications of recognizing that subjectivity plays a role in claims, again particularly in those that are less justified.

Finally, two participants when in 2nd grade study gave responses that seem to recognize the need to gather and provide information from different sources. Although some of their suggestions are not likely realistic forms of evidence (getting the answers from the creature), and other suggestions mention what might be good sources without specifically mentioning the evidence they would get, it is clear that they know that claims are not self-evident and information must be generated to justify a claim. For example, P99 said:

“I can take the creature to the house, to the kingdom and then I can test it, the hands, maybe if he speaks our language – I can also ask him. And just bring it and look at his hands. You can also go outside sometimes and look at what he’s doing with them.”

P141 said:

“I’ll try to talk to him and ask him’, Where did you come from? Are you a monster? Why did you come here?’ … I think maybe because he has such pink red spots he eats raspberries… [says to the experimenter] Go back to the picture of the creature… Yes, he eats things from the tree. And I think he’s green because he’s eating cucumbers and green things like lettuce… I’ll look for him and ask him all these questions. And if he does not know how to speak, then I’ll call my advisors to tell me how they understand him when he talks, that’s all.”

Although some of this participant’s comments might be seen to be speculative, based on limited observation, and off the topic of knowing why the creature has big hands and sharp claws, the participant offers evidence from claims for the creature’s coloring and suggest different acceptable sources of evidence, the creature’s testimony, observation, and the experts’ testimony.

Although none of the examples above show much understanding of either justification or evidence, it should be noted that the participants were not asked directly to produce either. What are seen are inklings of understanding that information and reliable sources need to be offered to justify claims to oneself and others. This stands in contrast to those with low iToM not quoted who offered no justification, repeated the explanations of the experts, or gave made up, irrelevant information or means of knowing (e.g., “you could use a magic mirror”).

## General Discussion

The longitudinal analysis of qualitative interviews provides support for two of the assumptions tested in the larger research project. For one, it has been assumed that the underlying task of epistemic development is coordinating the objective and subjective aspects of knowing. For this task to be approached, someone must appreciate the existence of and come to identify what might be subjective in knowing. In this research, we see that children are beginning this work. While some of the children at first, in 2nd grade, did not recognize conflicts in claims or noted them without being able to explain them, 2 years later children seem to have started work on this issue. By 4th grade, at the age of 9–10, all suggested that the difference in claims had some subjective reason, at least in differences of perspective and general ways of thinking if not in more specific internal cognitive or motivational processes. Whereas in 2nd grade, some suggested differences might be attributed to lack of knowledge, which could be easily remedied, by 4th grade the participants were pointing more toward differences between people.

Interestingly, at this level some of the participants emphasized more the subjective side of things, such as focusing on opinion or preference, while others hewed more closely to the objective side considering differences in emphasis on information with expertise. This might have been because at this age, children might still have absolutist views that ultimately one can find a single correct, objective answer. With this, the results provide some description of how children consider subjectivist aspects of knowing at the absolutist level which then may lead to the transition to multiplism. Alternatively, perhaps this difference is a precursor of what is seen in later development, such as with different types of multiplism with some holding that all perspectives are hopefully idiosyncratic and others holding that claims can be justified, but the multiplicity of perspectives is an impediment (Perry, 1970; Weinstock and Cronin, 2003). Also, in evaluativism there are objective relativists, for whom perspective differences lie with methodological differences and emphases on different evidence, and conceptual relativists, for whom perspective differences lie with systematic subjective contexts and individual interpretations (Leadbeater and Kuhn, 1989). This split in emphases on the objective or subjective aspects of knowing at different levels of epistemic development suggests it might be worthwhile to investigate cognitive, cultural, or individual factors in people’s orientations toward objectivity or subjectivity.

The second assumption of this research project supported by the results is that the development of iToM is significant in the turn toward grappling with subjectivism in knowing, and thus, toward epistemic development toward relativism. This helps provide a more complete picture of the development of epistemic development through the lifespan and provides a link between development of ToM and continuing developments of theories of knowledge. However, although the meaning of iToM is that children recognize that different knowledge claims arise because people interpret information differently, there was little evidence in the children’s interviews that they attributed claim differences between the scientists or in general to interpretation. Perhaps iToM sets children on the course to recognized subjective differences in how people receive, process, and emphasize information but the understanding of how interpretations arise and are used in knowledge building only comes later in epistemic development. This would be consistent with Kuhn and Weinstock’s (2002; see Barzilai and Weinstock, 2015) model of epistemic development which puts understanding that knowledge is interpretation at the heart of evaluativist thinking.

Whereas the research shows considerations of the meaning of perspective in knowing, it also points to fuzzy understanding of evidence and no real reference to how it relates to theoretical explanations. In some sense, this is not surprising given that young children struggle with differentiating evidence from explanation (Kuhn and Pearsall, 2000), and even adolescents who can distinguish between them find it easier to produce explanations (Glassner et al., 2005). However, the children in this study seemed more oriented toward the generation of evidence than toward producing explanations. Except for P180, who said that experts would explain things differently, they spoke little about explanation as something constructed. They seemed to expect that evidence and the gathering of information would point to one explanation or another. The search for evidence—such as talking to or directly observing the creature—would confirm the claim of one of the scientists. Good evidence is equated with first-hand observation. Thus, it seems that theory-evidence coordination is not supported by the epistemic understandings at this age.

In their seminal article on the understanding of the NOS, Carey and Smith (1993) distinguish between the two epistemologies of “knowledge unproblematic” and “knowledge problematic.” Although most of the participants’ expressions would seem to fall in the knowledge unproblematic category, that correct knowledge can be known certainly and that opinions might explain different claims, perhaps we do see hints of the progression toward the knowledge problematic. For instance, P180 in mentioning that how one thinks depends on one’s knowledge, expertise, and “angle” when looking at something, suggests a nascent understanding of interpretive frameworks. P249, who in 2nd grade gives the knowledge unproblematic response that people disagreed because they had different opinions without explanation, by 4th grade explains that people might draw different conclusions from the same perceptual experiences, a characteristic of the knowledge problematic, in saying that people can “discover two different things from the same part of a picture.” As if he had read Carey and Smith’s article, P249 goes on to say, “That implies one does not always come to an exact answer. This is problematic.”

It is likely that children, at least in 4th grade, do have a better understanding of how scientists investigate more than is apparent in the interviews, and that such understanding is based on epistemic understanding (Osterhaus et al., 2017). It is possible participants did not recognize the scientific issue in this study in the same way that they understand formal science in a school context. Aside from the context, the presentation of science issues through discrepant claims, as opposed to as open or answered questions, is not the common way of talking about science in the academic context. In one study (Tabak et al., 2010), biology undergraduates reported that they had not been exposed to discrepant claims as part of their coursework, in contrast with history undergraduates who reported that working through discrepant claims was a central part of their coursework. In addition, it is not too surprising that children do not have a sense of how justification in science takes place, other than through observation, when adolescents and many adults also are not particularly capable in distinguishing between the quality of science arguments which may or may not have reflected the differentiation of theory and evidence (Barchfeld and Sodian, 2009).

The qualitative analysis exploring and describing aspects of epistemic development suggests hypotheses regarding epistemic and NOS development that could be tested with quantitative studies. One, which was referred to earlier, is an analysis underway investigating whether iToM development predicts dimensions of epistemic thinking regarding the source, structure, and justification of knowledge (Weinstock, 2018). This research can be further developed to look at developments found in the current study between the 2nd and 4th grade in understanding disagreements and how knowledge is gained are reflected in the dimensions of source, structure, and justification of knowledge. In specific epistemic understanding of NOS, research could look at whether the development of iToM is a factor in explaining the trajectory of development found in research looking at the understanding of experiments (Osterhaus et al., 2017), theorizing (Metz, 2004; Koerber et al., 2015), and the coordination of theory and evidence (Kuhn et al., 2008). The current study did not reveal great understanding of hypothesis testing or the distinction between hypothesis and evidence—for instance, in response to the question about the distinction between guessing and knowing, even among those with high iToM the term hypothesis was used in relation to the term guess. Nevertheless, it would be worthwhile to examine whether the early understanding of perspective and interpretation found in iToM would be related to whether claims made from different perspectives might be seen as hypotheses which could be tested and if there is understanding of how evidence could be generated and brought to bear in testing such hypotheses.

Whereas we have looked at epistemic understanding as an outcome of development, it is important to note that we are not proposing that epistemic understanding, and the consequent understanding of knowledge in science and other disciplines, will come as a matter of course. Education, beyond age, has been found to be a factor in epistemic development (King and Kitchener, 1994; Kuhn et al., 2000; Tabak et al., 2010). There has been little research on how formal education might influence ToM, and particular iToM development, but there is evidence that mothers’ talk can promote earlier development of ToM (Ruffman et al., 2002) and iToM (Tafreshi and Racine, 2016), and that parents’ expression of epistemic information and their epistemic perspectives impact on their children’s evidence talk (Luce et al., 2013). Thus, how young children grapple with issues of epistemology and develop epistemic understandings, as have been found in this research, should help contribute to educational projects in the everyday understanding of science and how knowledge in general is constructed and justified.

## Data Availability Statement

The datasets generated for this study are available on request to the corresponding author.

## Ethics Statement

The studies involving human participants were reviewed and approved by the Chief Scientist’s Office, Israeli Ministry of Education and Ben-Gurion University of the Negev Human Subjects Research Committee. Written informed consent to participate in this study was provided by the participants’ legal guardian/next of kin and agreed to by the child participant.

## Author Contributions

MW is the PI of the research project. He planned the research, led the development of all of the research materials, worked with each of his collaborators on the coding and analyses of the data, and was the lead author on the manuscript. VI led the qualitative analysis across the different timepoints of the study. This analysis and the manuscript are based in part on her MA thesis. HC led the qualitative analysis in the first year of the study. The analysis and the manuscript are based in part on her MA thesis. IT worked with VI and HC in developing the coding scheme and analysis of the interviews. YH led the implementation of the research. Along with the PI, she led the development of all the research materials. She led the coding of the interpretive theory of mind task.

## Conflict of Interest

The authors declare that the research was conducted in the absence of any commercial or financial relationships that could be construed as a potential conflict of interest.
